# Immunogenicity and Protection of mRNA Vaccine Encoding Spike Protein of SARS-CoV-2 Omicron-XEC Subvariant

**DOI:** 10.3390/ijms27104218

**Published:** 2026-05-09

**Authors:** Xiaoqing Guan, Hansam Cho, Qian Liu, Shengnan Qian, Lanying Du

**Affiliations:** Institute for Biomedical Sciences, Georgia State University, Atlanta, GA 30303, USA

**Keywords:** coronavirus, COVID-19, SARS-CoV-2, Omicron variant, spike protein, mRNA vaccine, cross-neutralizing activity, protective efficacy

## Abstract

The surface spike (S) protein of severe acute respiratory syndrome coronavirus 2 (SARS-CoV-2) is a key target for the development of Coronavirus Disease 2019 (COVID-19) vaccines. Nevertheless, the mutations in the S protein, particularly in its receptor-binding domain region, have resulted in a reduced or complete loss of immunogenicity and/or protective efficacy in early vaccines against the Omicron variant and subvariants. Accordingly, continuous efforts are required to develop effective vaccines against multiple Omicron subvariants to reduce current and future threats. In this study, we designed an mRNA vaccine targeting the S protein of a recent Omicron-XEC subvariant (XEC-S-mRNA) and assessed its immunogenicity, including its broad neutralizing activity, and its protective efficacy against multiple Omicron subvariants. Our results demonstrated that the lipid nanoparticle-formulated mRNA vaccine formed an appropriate particle size with strong stability and successful antigen expression. It elicited durable cellular immune responses and broad neutralizing antibodies against multiple early and recent Omicron subvariants, thereby cross-protecting transgenic mice from challenge with a heterologous Omicron strain (KP.3). Moreover, the vaccine-induced neutralizing antibodies alone were sufficient to prevent Omicron-KP.3 infection. Overall, this study shows promise for further development of the candidate vaccine against current and future Omicron infections.

## 1. Introduction

Severe acute respiratory syndrome coronavirus 2 (SARS-CoV-2), the causative agent of the Coronavirus Disease 2019 (COVID-19) pandemic, was first detected in late December 2019 [1,2]. SARS-CoV-2 belongs to the same beta-genus coronaviruses as SARS-CoV and Middle East respiratory syndrome coronavirus (MERS-CoV), two other highly pathogenic coronaviruses, but it has much higher human-to-human transmissibility, with more infection and greater overall death tolls [3,4,5]. As of 5 April 2026, more than 779.2 million individuals have been infected with SARS-CoV-2, leading to over 7.1 million deaths [6]. SARS-CoV-2 continues to be a significant human health concern with consistent infections and an increasing number of cases, demonstrating a continual need for the development of safe and effective vaccines against virus infection.

The SARS-CoV-2 genome encodes four structural proteins, among which the surface spike (S) protein plays a key role in receptor binding and membrane fusion [7,8]. The receptor-binding domain (RBD) region in the S1 subunit of the S protein binds to the cellular receptor angiotensin-converting enzyme 2 (ACE2) to initiate viral infection [9,10,11,12]. Therefore, the S protein and its RBD region are crucial targets for vaccine development. In contrast to SARS-CoV and MERS-CoV, SARS-CoV-2 has undergone numerous mutations, deletions, or substitutions, particularly in the RBD region and/or the N-terminal domain of the S protein [13,14,15]. These mutations determine five major variants of concern strains (i.e., Alpha, Beta, Gamma, Delta, and Omicron) and multiple subvariants within the Omicron variant, including the recent XFG, XEC, and NB.1.8.1 subvariants [14,16,17,18,19]. Amino acid changes in the RBD residues of the Omicron variant and subvariants have shown significant effects in reducing the efficacy of vaccines that target the original strains and early variants of SARS-CoV-2 [20,21,22,23]. In this regard, vaccines with broad-spectrum neutralizing activity and cross-protective efficacy would have great potential for providing immunity against multiple Omicron subvariants of SARS-CoV-2 that could cause current and/or future epidemics or pandemics.

mRNA technology has created a convenient approach for the rapid development of safe and effective vaccines, thereby reducing the cost of large-scale vaccine production [24,25,26]. In this study, we constructed an mRNA vaccine encoding the S protein of the SARS-CoV-2 Omicron-XEC subvariant (XEC-S-mRNA) and evaluated its immunogenicity, including its broad neutralizing activity against early and recent Omicron subvariants, and its cross-protective efficacy in a murine model.

## 2. Results

### 2.1. Construction and Characterization of the XEC-S-mRNA Vaccine

**Figure 1:**
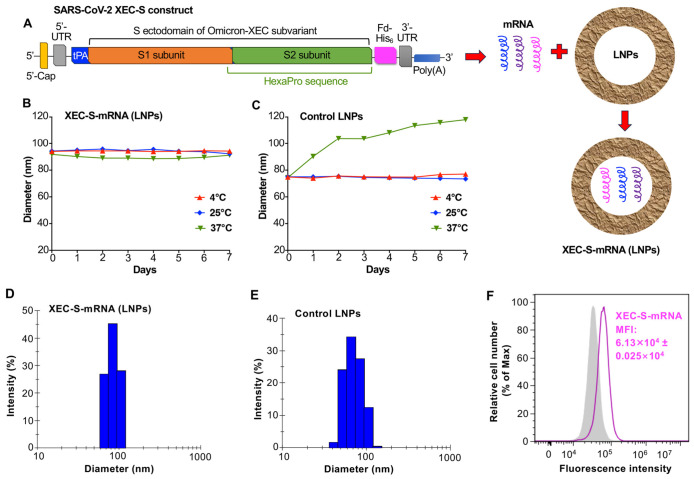
Construction and characterization of the XEC-S mRNA vaccine. (**A**) The XEC-S mRNA was constructed to encode the ectodomain S (S1 and S2 subunits) protein of the Omicron-XEC subvariant of SARS-CoV-2 with the HexaPro sequence, and to contain an N-terminal tissue plasminogen activator (tPA signal peptide), a C-terminal foldon trimeric sequence and a His_6_ tag. The synthesized mRNA carrying a 5′-untranslated region (5′-UTR) and a 3′-UTR was capped at the 5′-terminus and tailed with a poly(A) sequence at the 3′-terminus, then encapsulated with lipid nanoparticles (LNPs) to form XEC-S-mRNA LNPs. Measured stability of the LNP-formulated XEC-S-mRNA (**B**) and control LNPs (**C**) by a DynaPro NanoStar II Light Scattering Detector (DLS) instrument. The samples were stored at 4 °C, 25 °C, and 37 °C for 1 to 7 days, then the particle sizes (diameters) were measured by the DLS. Histograms showing particle sizes of the LNP-formulated XEC-S-mRNA (**D**) and control LNPs (**E**). (**F**) Assessment of the expression of the His-tagged protein encoded by XEC-S-mRNA using flow cytometry. HEK293T cells were incubated with XEC-S mRNA-LNPs or the control LNPs, then stained with the anti-His-FITC antibody prior to conducting fluorescence intensity analysis using a flow cytometer. The shaded region indicates control cells incubated with LNPs, and the magenta line refers to target cells incubated with LNP-formulated XEC-S-mRNA. MFI: median fluorescence intensity.

The XEC-S-mRNA vaccine was constructed to encode the ectodomain of the S protein of Omicron-XEC with a HexaPro sequence, an N-terminal tissue plasminogen activator (tPA) signal peptide, a C-terminal foldon trimeric motif and a His_6_ tag, followed by synthesis using a T7 transcription kit (Figure 1A). The synthesized mRNA was capped at the N-terminus, tailed with a poly(A) at the C-terminus, and then encapsulated with lipid nanoparticles (LNPs) to increase its stability during delivery to target cells or animals (Figure 1A).

The particle size and stability of the LNP-encapsulated XEC-S-mRNA at different temperatures were characterized using a DynaPro NanoStar II Light Scattering Detector (DLS) instrument. Overall, this mRNA was stable at all temperatures tested (i.e., 4 °C, 25 °C, and 37 °C) during the 7-day test period, with a particle size between 88 and 96 nm (Figure 1B). By comparison, the LNPs alone, without mRNA, had a slightly decreased particle size, and were not stable at 37 °C (Figure 1C). Similar sizes of XEC-S-mRNA encapsulated in LNPs and the LNP-alone control are shown in the histogram figures (Figure 1D,E). Flow cytometry analysis further identified the protein expression of His_6_-tagged XEC-S-mRNA in target HEK293T cells (Figure 1F). These data confirm the stability and successful antigen expression of the synthesized XEC-S-mRNA vaccine.

### 2.2. XEC-S-mRNA Vaccine-Induced Potent Antibody Responses with Broad Neutralizing Activity

**Figure 2:**
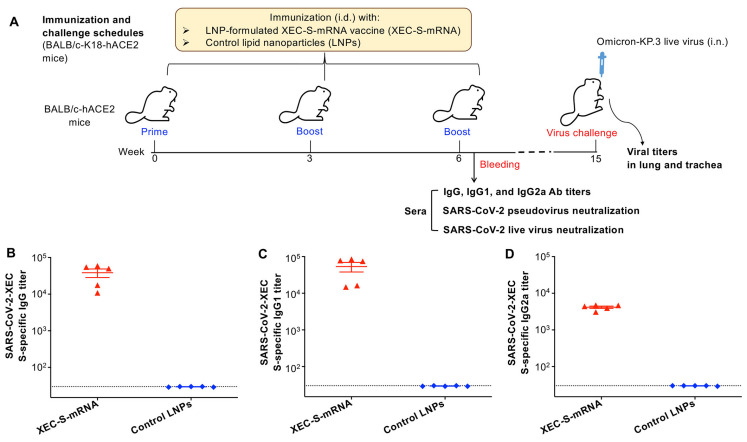
Assessment of humoral immune responses induced by the XEC-S-mRNA vaccine. (**A**) Immunization and challenge schedules. BALB/c-hACE2 transgenic mice were intradermally (i.d.) immunized with LNP-formulated XEC-S-mRNA or control LNPs and boosted twice at 3-week intervals; collection of sera followed 10 days after the last dose for measurement of subsequent antibody responses. Nine weeks after the last dose, the immunized mice were then intranasally (i.n.) challenged with an Omicron-KP.3 subvariant of SARS-CoV-2 to assess the protective efficacy. Evaluation of the XEC-S-specific IgG (**B**), IgG1 (**C**), and IgG2a (**D**) antibody (Ab) titers in sera by ELISA. The data refers to the mean ± standard deviation of the mean (s.e.m) of five mice in each group. The dotted lines indicate the detection limit (1:30). The experiments were repeated once, with similar results obtained.

To investigate the humoral immune responses induced by the XEC-S-mRNA vaccine, BALB/c-based K18-hACE2 (hereinafter BALB/c-hACE2) transgenic mice were intradermally (i.d.) immunized with either the LNP-encapsulated XEC-S-mRNA or the LNP control three times at 3-week intervals (based on our previously optimized protocol) [27]. Sera collected 10 days after the last dose were tested for S-specific antibody responses and neutralizing activity against multiple recent Omicron subvariants of SARS-CoV-2 (Figure 2A).

As expected, high-titer IgG antibodies specific to the S protein of Omicron-XEC were observed in the XEC-S-mRNA-immunized mice (Figure 2B). The XEC-S-mRNA also induced similarly high-titer S-specific IgG1 subtype (Figure 2C) and favorable IgG2a subtype (Figure 2D) antibodies.

**Figure 3:**
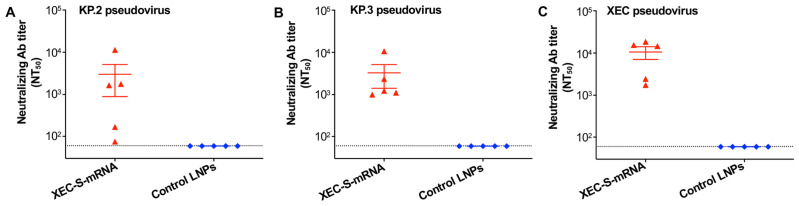

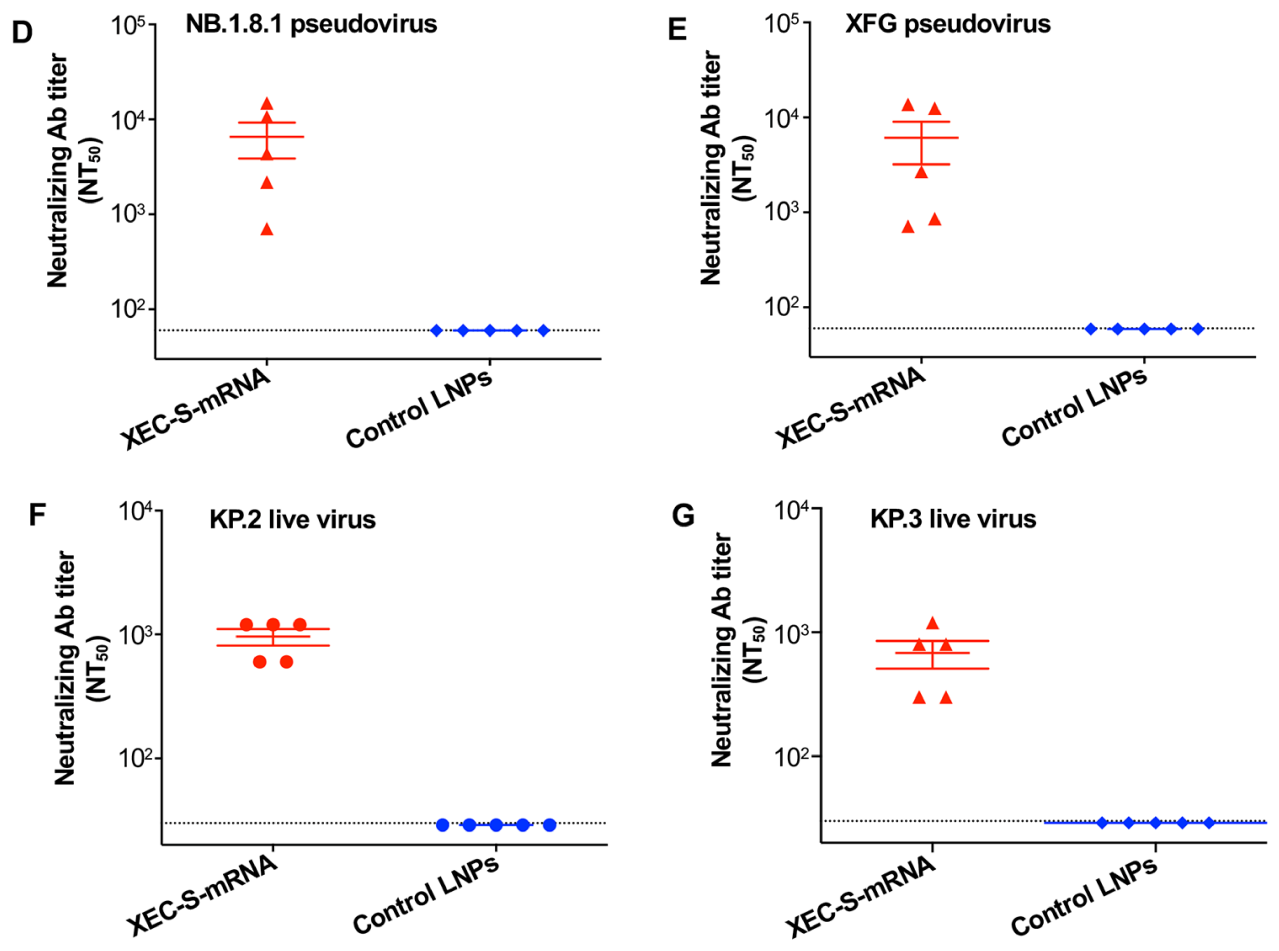
Evaluation of the broad neutralizing antibody responses induced by the XEC-S-mRNA vaccine. Mouse sera collected 10 days after the third immunization were assessed for a neutralizing antibody (Ab) titer against pseudotyped Omicron-KP.2 (**A**), KP.3 (**B**), XEC (**C**), NB.1.8.1 (**D**), and XFG (**E**) using a pseudovirus neutralization assay. The same sera were assessed for a neutralizing Ab titer against the infection of live SARS-CoV-2 Omicron subvariants, including KP.2 (**F**) and KP.3 (**G**), using a cytopathic effect (CPE)-based neutralization assay. The NT_50_ (i.e., 50% neutralizing Ab titer) is shown as the mean ± s.e.m of five mice in each group. The dotted lines indicate the detection limit (1:60 for the pseudovirus neutralizing Ab titer, and 1:30 for the live virus neutralizing Ab titer). The experiments were repeated once, with similar results obtained.

These antibodies potently neutralized the infection of all the pseudotyped Omicron subvariants of SARS-CoV-2 tested, including the early and recent epidemic strains of KP.2, KP.3, XEC, NB.1.8.1 and XFG (Figure 3A–E). The neutralizing activity of the serum was confirmed by a live virus neutralization assay to neutralize the KP.2 and KP.3 strains (Figure 3F,G). These data indicate the ability of the XEC-S-mRNA vaccine to elicit highly potent and broad neutralizing antibodies against multiple strains of the SARS-CoV-2 Omicron variant.

### 2.3. The XEC-S-mRNA Vaccine Protected Transgenic Mice from the Challenge of SARS-CoV-2 Omicron-KP.3

**Figure 4:**
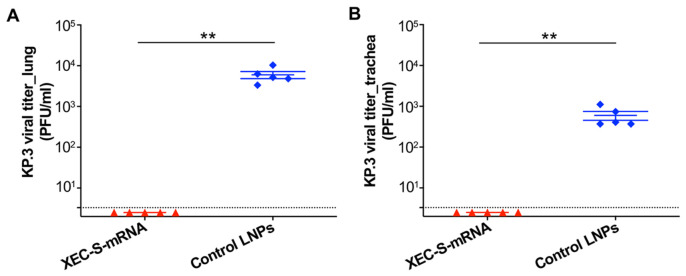
The LNP-formulated XEC-S-mRNA vaccine protected against a SARS-CoV-2 Omicron-KP.3 challenge. Nine weeks after the final immunization, the BALB/c-hACE2 transgenic mice were challenged (i.n.) with an Omicron-KP.3 subvariant of SARS-CoV-2, then viral titers in the lungs (**A**) and trachea (**B**) were measured by means of the plaque assay 5 days post-challenge. The data (plaque-forming unit: PFU/mL of viral titers) is shown as the mean ± s.e.m of five mice in each group. The dotted lines indicate the detection limit (3.3 PFU/mL). The unpaired Student’s *t* test was used to analyze statistical significance between the XEC-S-mRNA and control LNP groups. ** indicates *p* < 0.01. The experiments were repeated once, with similar results obtained.

The protective efficacy of XEC-S-mRNA was assessed in the immunized BALB/c-hACE2 transgenic mice 9 weeks after the last vaccination. The mice were intranasally (i.n.) challenged with the Omicron-KP.3 subvariant of SARS-CoV-2, and the viral titers in the lungs and trachea were evaluated 5 days post-infection (Figure 2A). Viral titers in the mice immunized with the XEC-S-mRNA vaccine were below the detection limit in both the lungs (Figure 4A) and the trachea (Figure 4B), and were significantly lower than those in the control mice receiving only LNPs. These data demonstrate the efficacy of the XEC-S-mRNA vaccine in cross-protecting mice against an Omicron-KP.3 challenge.

### 2.4. Antibodies Induced by the XEC-S-mRNA Vaccine Played a Key Role in Protecting Against a SARS-CoV-2 Challenge

**Figure 5:**
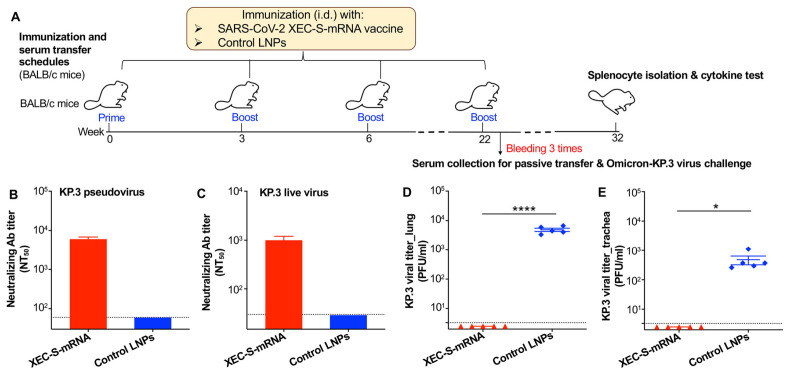
XEC-S-mRNA-induced neutralizing antibodies play a key role in the protection against a SARS-CoV-2 Omicron-KP.3 challenge. (**A**) Immunization and serum transfer schedules. BALB/c mice were immunized (i.d.) with LNP-formulated XEC-S-mRNA or control LNPs and boosted at 3, 6, and 22 weeks. The pooled sera collected at 10, 17, and 28 days after the last dose were assessed for neutralizing antibody (Ab) titers against pseudotyped (**B**) and live (**C**) Omicron-KP.3 subvariants of SARS-CoV-2, then injected (i.p.) into naïve B6-hACE2 transgenic mice. 6 h post serum-transfer, the mice were challenged (i.n.) with Omicron-KP.3; five days post-challenge, the lungs (**D**) and trachea (**E**) were collected and assessed for viral titers using the plaque assay. The NT_50_ indicates a 50% neutralizing Ab titer, and the viral titer is expressed as PFU/mL. The data is shown as the mean ± s.e.m of duplicate or quadruple wells (for the pooled sera) or of five mice in each group (for the viral titer). The dotted lines indicate the detection limit (1:60 for the pseudovirus neutralizing Ab titer, 1:30 for the live virus neutralizing Ab titer, and 3.3 PFU/mL for the viral titer). The unpaired Student’s *t* test was used to analyze the statistical significance between the XEC-S-mRNA and control LNP groups. * and **** indicate *p* < 0.05 and *p* < 0.0001, respectively. The experiments were repeated once, with similar results obtained.

In a separate experiment, BALB/c mice were immunized with either the XEC-S-mRNA vaccine or LNP control and boosted at 3 and 6 weeks (Figure 5A). To determine whether XEC-S-mRNA-induced neutralizing antibodies were important in protecting against SARS-CoV-2 Omicron infection, the mice were boosted again at 22 weeks (with the goal of obtaining high-titer serum neutralizing antibodies for challenge studies), and the pooled sera were intraperitoneally (i.p.) injected into naïve C57BL/6-based K18-hACE2 (B6-hACE2) transgenic mice (with the goal of validating the protective efficacy in another mouse strain) (Figure 5A). This was followed by the challenge of the B6-hACE2 mice with the Omicron-KP.3 subvariant 6 h post-serum injection, with measurement of viral titers in the lungs and trachea performed 5 days after the challenge (Figure 5A). Indeed, the pooled sera from the XEC-S-mRNA-immunized BALB/c mice exhibited potent neutralizing activity against infection from both the pseudotyped (Figure 5B) and live virus (Figure 5C) of the Omicron-KP.3 subvariant of SARS-CoV-2. As expected, B6-hACE2 mice receiving these immune sera presented significantly lower viral titers in the lungs (Figure 5D) and trachea (Figure 5E) compared to B6-hACE2 mice receiving sera from BALB/c mice injected with control LNPs alone (Figure 5), with the former values both being below the detection limit. These results show that serum antibodies elicited by the XEC-S-mRNA vaccine are critical for passively protecting naïve mice from a SARS-CoV-2 Omicron-KP.3 challenge.

### 2.5. The XEC-S-mRNA Vaccine Induced Durable Cellular Immune Responses

**Figure 6:**
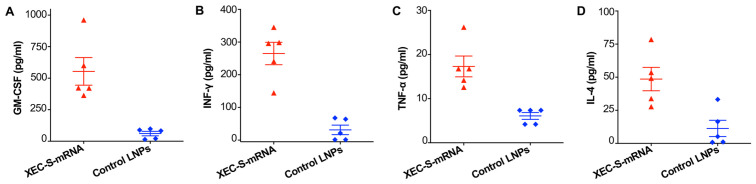
Assessment of cellular immune responses induced by the XEC-S-mRNA vaccine. 10 weeks after the final immunization with LNP-formulated XEC-S-mRNA or control LNPs, the spleen of BALB/c mice were collected and S-specific cytokines, including GM-CSF (**A**), IFN-**γ** (**B**), TNF-α (**C**), and IL-4 (**D**), were measured in the isolated splenocytes using the multiplex assay. The data is shown as the mean ± s.e.m of five mice in each group. The experiments were repeated once, with similar results obtained.

The ability of the XEC-S-mRNA vaccine to induce durable cellular immune responses was evaluated in immunized BALB/c mice 32 weeks after the immunization, and splenocytes were isolated for this test using a multiplex assay (Figure 5A). Notably, high-levels of GM-CSF, IFN-γ, TNF-α, and IL-4 cytokines were observed in the mice immunized with the XEC-S-mRNA, whereas only background levels of such cytokines were found in the control mice receiving LNPs alone (Figure 6A–D). These data reveal that the XEC-S-mRNA elicited effective and long-term cellular immune responses in the immunized mice.

## 3. Discussion

The Omicron variant of SARS-CoV-2 emerged in 2021, and has led to dozens of subvariants, including early strains XBB.1.5, JN.1, KP.2, and KP.3, and recent strains XFG, NB.1.8.1, and XEC [4,28,29,30]. Continuous human infections, particularly from recent SARS-CoV-2 Omicron subvariants, demonstrate the necessity for the development of efficacious and safe vaccines to fight against infection from multiple Omicron subvariants, recent strains in particular, and to reduce current and future threats.

Because of its crucial role in viral infection and pathogenesis, the S protein of SARS-CoV-2 is considered to be the major target for the development of effective COVID-19 vaccines [31,32]. Various vaccines have been developed targeting the S protein of SARS-CoV-2 [32,33,34,35,36]. The neutralizing activity and/or protective efficacy of most S-based vaccines targeting the original and early variants of SARS-CoV-2 have been significantly reduced or completely lost against Omicron variants, owing to the numerous substitutions in the RBD region of these strains [37,38,39,40].

Several vaccine types have been developed against the Omicron variant and its subvariants of SARS-CoV-2, including vaccines based on mRNAs, viral vectors, subunit proteins, nanoparticles, and mRNA virus-like particles [41,42,43,44,45]. For example, a parainfluenza virus-vectored vaccine encoding the S protein of Omicron-B.1.1.529 elicited mucosal IgA and systemic antibody responses in hamsters, providing homologous and heterologous protection [46]. Notably, the combination of two different vaccine types, such as an adenovirus vectored vaccine encoding the S protein of Omicron-XBB.1.5, and a subunit vaccine expressing a trimeric RBD of Omicron subvariants, induces robust immune responses against Omicron subvariants of SARS-CoV-2 [43,47]. In contrast to traditional vaccine types, mRNA vaccines have the advantages of fast production, quick scaling with cost effectiveness, easy adaptability to new variants, high efficacy, and a strong safety profile without the risk of DNA integration or alteration [48,49,50]. Therefore, at least two mRNA vaccines were rapidly approved during the early stages of the COVID-19 pandemic to prevent viral spread and improve survival [51,52,53].

Determining the immunogenicity and protection of vaccines based on the recent Omicron subvariants against multiple early and current strains of SARS-CoV-2 remains crucial. Here, we designed a unique mRNA vaccine targeting the S protein of the recent Omicron-XEC subvariant of SARS-CoV-2 (XEC-S-mRNA), tested its broad neutralizing activity against early KP.2 and KP.3 strains, as well as against recent XFG, XEC, and NB.1.8.1 strains, and evaluated its protective efficacy against an Omicron-KP.3 challenge. This mRNA vaccine formed an appropriate particle size after encapsulation with LNPs and was stable for at least 7 days at different temperatures, including at 25 °C and 37 °C. Based on our prior studies, three-dose immunization regimens of SARS-CoV-2 mRNA vaccines elicited broader and better neutralizing antibodies against different variants than one-dose or two-dose administrations [27,54]. Therefore, the immunization schedule in the current study employed this optimal protocol. Notably, the XEC-S-mRNA elicited potent cross-neutralizing antibodies against all early and recent Omicron subvariants tested, with cross-protective efficacy against the Omicron-KP.3 strain. Importantly, vaccine-induced neutralizing antibodies play a critical role in protecting naive mice against a KP.3 challenge, although the XEC-S-mRNA also elicited durable, KP.3-specific cellular immune responses.

It is shown that the XEC-S-mRNA-encoded protein had detectable antigen expression in target HEK293T cells, demonstrating a successful vaccine design. The efficiency of this mRNA vaccine could be further improved through optimization of the S antigen by adjusting its length, sequences, or codon usage; maximization of the mRNA component performance or package systems, including nucleotide or UTR sequence modification, or capping and tailing processing, to increase the long-term stability and translation efficiency; and improvement of formulations, by assessing diverse LNPs, or a change in delivery approaches, such as targeted delivery of the mRNA to specific tissues or immune cells [48,55,56,57,58]. These optimizations would offer the potential to enhance the overall immunogenicity and/or protective efficacy of the mRNA vaccines [48,49,59]. Another limitation of the study is that the vaccine-induced cellular immune responses were evaluated by a multiplex assay, which showed increased cytokine secretion, but the exact source of cytokines (CD4^+^ or CD8^+^ T cells) was not identified. Future studies would be warranted to assess whether CD4^+^ or CD8^+^ T cells are the primary producers of antigen-specific cytokines, to evaluate the vaccine-induced long-lasting and durable memory T-cell responses, and to identify the roles of T cells in protecting against infection from recent SARS-CoV-2 Omicron subvariant(s).

## 4. Materials and Methods

### 4.1. Cell Culture

HEK293T cells expressing SARS-CoV-2 receptor human ACE2 (hACE2/293T, Laboratory stock) and HEK293T cells (ATCC, Manassas, VA, USA) were diluted in Dulbecco’s Modified Eagle Medium (DMEM) cell culture medium containing 1% Penicillin-Streptomycin solution (Corning, New York, NY, USA) and 10% Fetal Bovine Serum (FBS) (R&D Systems, Minneapolis, MN, USA), and cultured in a 37 °C cell culture incubator supplied with 5% CO_2_. HEK293F cells (Thermo Fisher Scientific, Waltham, MA, USA) were diluted in a serum-free medium (ESF SFM) (Expression Systems, Davis, CA, USA) containing 1% Antibiotic-Antimycotic solution (Corning), and cultured in a 37 °C cell culture incubator supplied with 8% CO_2_ and subjected to constant rotation at 120 rpm.

### 4.2. Mice

Male and female BALB/c mice, BALB/c mice expressing human ACE2 (i.e., BALB/c-K18-hACE2 or BALB/c-hACE2), and C57BL/6 mice expressing human ACE2 (i.e., B6-K18-hACE2 or B6-hACE2) were initially obtained from the Jackson Laboratory, then bred in our animal facilities until use. The mice were randomly allocated to various groups (5 mice in each group) for the immunization and/or challenge experiments described below.

### 4.3. Construction of the mRNA Vaccine

The template for mRNA synthesis was designed and constructed as described below. The S gene of the SARS-CoV-2 Omicron-XEC subvariant (GISAID number: EPI_ISL_19612171) was codon-optimized and synthesized by GenScript (Piscataway, NJ, USA), which consists of a HexaPro sequence (e.g., the furin cleavage site mutation and six proline amino acid substitutions). The synthesized XEC-S gene, together with an N-terminal signal peptide (tPA), a C-terminal trimeric foldon and 6× His tag sequences, was amplified via PCR and ligated with an enzyme-digested pCAGGS expression vector using Seamless Cloning reagent (Vazyme International LLC, Woburn, MA, USA). A positive XEC-S recombinant plasmid was verified by Sanger DNA sequencing (Psomagen, Rockville, MD, USA).

### 4.4. mRNA Synthesis

The mRNA vaccine was synthesized as described below. After linearizing the aforementioned XEC-S recombinant plasmid, the mRNA was synthesized in vitro using a T7 Transcription Kit (MEGAscript™, Thermo Fisher Scientific), according to the manufacturer’s instructions. This was performed using a naturally occurring nucleotide, Pseudo-UTP (APExBIO, Houston, TX, USA), which incorporates pseudouridine as a substitute for uridine, together with three regular nucleotides, including ATP, GTP, and CTP. The ScriptCap™ Cap 1 Capping System (CELLSCRIPT, Madison, WI, USA) and an A-Plus™ Poly(A) Polymerase Tailing Kit (CELLSCRIPT) were then utilized to add the Cap1 structure and a poly(A) tail to the 5′ and 3′ ends of the synthesized mRNA, respectively. The mRNA products were purified using a Monarch^®^ Spin RNA Cleanup Kit (New England Biolabs, Ipswich, MA, USA) and stored at −80 °C until further use.

### 4.5. LNP Formulation with mRNA

The LNP was formulated by dissolving IM21.7c (PolyPlus, Berkeley, CA, USA), SM102 (Cayman Chemical, Ann Arbor, MI, USA), DSPC (Avanti Research, Foster, CA, USA), cholesterol (Sigma-Aldrich, St. Louis, MO, USA), and DMG-PEG2000 (Cayman Chemical) at a ratio of 20:30:10:38.5:1.5 in anhydrous ethanol. The above-synthesized mRNA was dissolved in 10 mM of sodium acetate (pH 4). The LNP and XEC-S mRNA were mixed at a volume ratio of 1:3 using a NanoAssemblr™ Ignite System (Cytiva, Marlborough, MA, USA) for nanoparticle formulation. The XEC-S mRNA-LNP formulation was then diluted with a 30-fold volume of 1× phosphate buffer saline (PBS) (pH 7.4) and concentrated with Amicon Ultra Centrifugal Filter (10 kDa MWCO; MilliporeSigma, Burlington, MA, USA). The particle size of the formulated mRNA-LNP was measured using the DLS Detector (Wyatt Technology, Santa Barbara, CA, USA).

### 4.6. Detection of mRNA Expression by Flow Cytometry

To test the expression of the His-tagged, mRNA-encoded protein, HEK293T cells were seeded onto a 6-well cell culture plate and cultured overnight until the cells reached 75% confluency. The cells were then incubated with either 4 µg of the XEC-S mRNA-LNPs or the control LNPs. After 24 h, Brefeldin A (Biolegend, SanDiego, CA, USA) was added to the cells at a final concentration of 2.5 µg/mL, with the goal to block the secretion of protein. The cells were continued to culture for another 24 h, followed by detachment with trypsin and two washes with 1× PBS. The cells were stained with eFluor 780 Fixable Viability Dye (Thermo Fisher Scientific) at 4 °C for 20 min, then fixed and permeabilized with a Cytofix/Cytoperm kit (BD Biosciences, Bedford, MA, USA). The fixed and permeabilized cells were further stained with an anti-His-FITC antibody (1:100 dilution; Thermo Fisher Scientific) at room temperature for 30 min. After the staining, the cells were analyzed for protein expression using a CytoFLEX flow cytometer (Beckman Coulter Life Sciences, Brea, CA, USA). The resulting data was analyzed using FlowJo software v7.6.

### 4.7. Preparation of Recombinant Protein

HEK293F cells were transfected by a sequence-verified recombinant His-tagged plasmid encoding S protein of a SARS-CoV-2 Omicron-XEC subvariant (e.g., XEC-S protein) using PEI MAX (Linear Polyethylenimine Hydrochloride, M.W 40,000) (Kyfora Bio, Horsham, PA, USA), with a DNA: PEI MAX weight ratio of 1:3. Five days after transfection, the culture supernatant containing the expressed protein was collected by centrifugation at 6000× *g* for 20 min. The expressed protein was purified using Ni-NTA Superflow Resin (Qiagen, Germantown, MD, USA), followed by dialysis against 1× PBS (pH 7.4) using Amicon Ultra Centrifugal Filter (50 kDa MWCO, MilliporeSigma).

### 4.8. ELISA

The titer of specific antibodies (IgG, IgG1, and IgG2a) in sera of the immunized mice was determined using the ELISA technique. Half-area, 96-well ELISA microplates were coated with the above-purified His-tagged XEC-S protein (1 μg/mL) in a 50 mM carbonate coating buffer (pH 9.4) overnight at 4 °C. The coated plates were washed once with 1× PBS containing 0.1% Tween-20 (PBST) and blocked with 2% fat-free milk (Bio-Rad, Hercules, CA, USA) prepared in PBST at 37 °C for 1 h. After washing with PBST, the plates were incubated with serially diluted mouse sera at 37 °C for 2 h. The plates were then washed again with PBST 6 times and incubated at 37 °C for 1 h with horseradish peroxidase (HRP)-labeled anti-mouse IgG antibody (1:25,000 dilution; Thermo Fisher Scientific), anti-mouse-IgG1 antibody (1:25,000 dilution; Thermo Fisher Scientific), or anti-mouse-IgG2a antibody (1:2000 dilution; Thermo Fisher Scientific). After washing 6 times with PBST, the plates were incubated with the substrate TMB (3,3′,5,5′-Tetramethylbenzidine) (MilliporeSigma), before terminating the reaction with 1 N of sulfuric acid. Absorbance at 450 nm (OD_450_) was acquired by reading the plates with a Cytation 7 microplate reader (BioTek Instruments, Winooski, VT, USA). The OD_450_ values were analyzed via non-linear regression, and the titer of specific antibodies was calculated accordingly (the cutoff was set as four times the blank (e.g., 0.192)).

### 4.9. SARS-CoV-2 Pseudovirus Generation and Neutralization Assay

The pseudoviruses, including the KP.2, KP.3, XEC, NB.1.8.1, and XFG Omicron subvariants of SARS-CoV-2, were produced from HEK293T cells co-transfected with pLenti-CMV-LUC (Addgene, Watertwon, MA, USA) plasmid, psPAX2 (Addgene) plasmid, and each of the recombinant plasmids (pcDNA3.1 backbone) encoding-relevant S protein of the above SARS-CoV-2 Omicron subvariants, using the PEI MAX transfection reagent. The medium was replaced with a fresh culture medium (e.g., DMEM containing 10% FBS and a 1% Penicillin-Streptomycin solution) ~18 h post-transfection. The culture supernatant containing the produced pseudovirus was harvested 2 to 3 days after transfection by centrifugation at 2000 rpm for 5 min. For the pseudovirus neutralization assay, target cells (hACE2/293T) were seeded onto 96-well culture plates (2 × 10^4^ cells/100 µL per well) 4~6 h before the experiment. The mouse sera at serial dilutions were pre-incubated with the respective types of pseudoviruses collected above at 37 °C for 1 h, then 100 µL/well of the serum-pseudovirus mixture was added to the above pre-seeded target cells in 96-well plates. After 24 h, the cells were replenished with 80 µL of fresh culture medium, then they continued to culture for another 48 h. The cells were lysed with cell lysis buffer (Promega, Madison, WI, USA) at room temperature for 1 h, followed by the addition of luciferase substrate (Promega). The relative luciferase activity was immediately measured via a Cytation 7 imaging reader (BioTek Instruments). The NT_50_ values (i.e., the serum dilution at which the pseudovirus was neutralized by 50%) were calculated as the pseudovirus neutralizing antibody titer using a method based on the Median Effect Principle.

### 4.10. SARS-CoV-2 Live Virus Neutralization Assay

A CPE-based neutralization method was used to evaluate neutralizing activity of the sera of mice immunized against KP.2 and KP.3 Omicron subvariants of live SARS-CoV-2. Target cells (Vero E6) were seeded onto 96-well culture plates 24 h before the experiment. The mouse sera at serial dilutions were pre-incubated with 100 TCID_50_ (50% tissue culture infectious dose) per well of each virus at 37 °C for 1 h, and the mixture of sera and virus was added to the above pre-seeded target cells in 96-well plates. The cells continued to culture at 37 °C for 4–5 days and were then assessed for the presence of CPE using a microscope. The NT_50_ values (e.g., the highest serum dilution at which at least 50% of the cells were inhibited from the CPE) were calculated as the live virus neutralizing antibody titer.

### 4.11. Mouse Immunization and Challenge Procedures

To evaluate the immune responses and protective efficacy induced by the mRNA vaccine, BALB/c-hACE2 transgenic mice (~20–24-week-old, female and male) were (i.d.) vaccinated with either the LNP-formulated XEC-S-mRNA vaccine (100 μL containing 10 μg mRNA per mouse) or control LNPs (100 μL per mouse) using an optimal three-dose scheme at three-week intervals. Ten days after the third injection, the sera were collected via facial bleeding and inactivated at 56 °C for 30 min before use. The immunized mice were (i.n.) challenged with the KP.3 live virus of the SARS-CoV-2 Omicron subvariant (10^4^ plaque-forming unit (PFU)/mouse) under anesthesia 9 weeks after the third dose of vaccination.

### 4.12. Sample Preparation and Passive Protective Efficacy

Immune sera for passive transfer were generated by immunizing ~9–11-week-old, male BALB/c mice with the LNP-formulated XEC-S-mRNA vaccine (10 μg/100 μL/mouse) or control LNPs (100 μL/mouse) via i.d. route at weeks 0, 3, 6 and 22. The serum samples were collected by facial bleeding 3 times (10, 17, and 28 days after the final immunization) and inactivated at 56 °C for 30 min. The pooled mouse immune sera were tested for neutralizing antibodies against infection from the Omicron-KP.3 pseudovirus and live virus. These pooled mouse immune sera were then passively transferred into ~10–12-week-old, naïve female and male B6-hACE2 mice (200 μL/mouse, 5 mice/group) via the intraperitoneal (i.p.) route. The mice receiving the pooled sera were further (i.n.) challenged with the KP.3 live virus of the SARS-CoV-2 Omicron subvariant (10^4^ PFU/mouse) 6 h post-transfer of the immune sera.

### 4.13. Viral Titer Detection in Challenged Mice

The mice challenged with the SARS-CoV-2 KP.3 virus described above were sacrificed on day 5 post-challenge, and lung and trachea tissue samples were collected for the measurement of viral titers using the plaque assay described below. The tissues were homogenized and added with serial dilutions to pre-seed Vero E6 cells, prior to incubation at 37 °C for 1 h. After removing the culture medium, an MEM culture medium containing 2% FBS and 1.25% carboxymethyl cellulose solution was added to cells, then the cells continued to culture at 37 °C for 4 days. After washing with PBS, the cells were fixed with 10% formaldehyde at 37 °C for 2 h and stained with 0.5% crystal violet, until the presence of plaques was observed. The viral titer in the tissues was calculated based on the number of plaques, which was shown as the PFU/mL of each tissue sample.

### 4.14. Splenocyte Cytokine Expression

A multiplex assay was used to detect cytokine expression in immunized mouse splenocytes in response to the XEC-S protein stimulation. Ten weeks after the final immunization of the BALB/c mice with the LNP-formulated XEC-S-mRNA or control LNPs described above, the mice were euthanized, and the splenocytes were extracted for evaluation of cellular immune responses via the multiplex assay. The splenocytes isolated from the immunized or control mice were cultured in a RPMI 1640 medium, which contained 1% Penicillin-Streptomycin solution, 10% FBS, 1 mM of sodium pyruvate, 55 µM of β-Mercaptoethanol, and 1% non-essential amino acids (Sigma-Aldrich). The splenocytes were seeded onto 96-well, round-bottom culture plates (1 × 10^6^ cells/mL, 200 µL/well) and stimulated with the XEC-S protein of SARS-CoV-2 (5 μg/mL) for four days in a 37 °C cell culture incubator supplied with 5% CO_2_. The cell culture supernatant was collected after centrifugation and cytokine expression was then measured using a Bio-Plex Pro Mouse Cytokine Assay kit (Bio-Rad). The results were analyzed with the Bio-Plex 200 System instrument (Bio-Rad).

### 4.15. Statistical Analysis

An unpaired-*t* test was used to compare difference between the two groups. *p* < 0.05, *p* < 0.01, and *p* < 0.0001 are shown as *, **, and ****, respectively. All the statistical calculations were performed using the statistical software GraphPad Prism 10.

## 5. Conclusions

In summary, the designed XEC-S-mRNA vaccine elicited durable cellular immune responses and highly effective and broad-spectrum neutralizing antibodies against multiple Omicron subvariants, with cross-protective efficacy against a heterologous Omicron strain. Thus, this mRNA vaccine shows promise for further development against current and future Omicron viral infections.

## Data Availability

The original contributions presented in this study are included in the article. Further inquiries can be directed to the corresponding author.

## Abbreviations

The following abbreviations are used in this manuscript:

ACE2	Angiotensin-converting enzyme 2
COVID-19	Coronavirus Disease 2019
LNPs	Lipid nanoparticles
MERS-CoV	Middle East respiratory syndrome coronavirus
PFU	Plaque-forming unit
RBD	Receptor-binding domain
S	Spike
SARS-CoV-2	Severe acute respiratory syndrome coronavirus 2
tPA	Tissue plasminogen activator
